# Smart Products in Livestock Farming—An Empirical Study on the Attitudes of German Farmers

**DOI:** 10.3390/ani11041055

**Published:** 2021-04-08

**Authors:** Sirkka Schukat, Heinke Heise

**Affiliations:** Department of Agricultural Economics and Rural Development, University of Göttingen, 37073 Göttingen, Germany; heinke.heise@agr.uni-goettingen.de

**Keywords:** cluster analysis, farmers’ attitudes, smart farming, smart livestock farming, smart products

## Abstract

**Simple Summary:**

In the last few years, the fourth industrial revolution has found its way into agriculture. Under the heading of smart farming, various so-called smart products are offered that can positively influence the daily work of farmers as well as animal welfare. These smart products can record data from the farming operation, extract essential information, and in some cases even make decisions autonomously. Particularly in Germany, where social criticism of intensive livestock farming has been raised, such smart products could make a significant contribution to improving animal welfare. However, a key prerequisite is the acceptance of the users, who are typically the livestock farmers themselves. There is so far hardly any knowledge about farmers’ attitudes towards smart products in livestock farming. In this study, the attitudes of German livestock farmers towards smart products are evaluated by categorizing them into groups by a factor analysis and a cluster analysis. Based on the analysis of an online questionnaire, in which German livestock farmers (n = 422) participated, four clusters could be derived. The main distinguishing characteristics of the clusters are the influence of the social environment, the expected effort for implementation, the general trust in smart products, and the technology readiness of the farms. As a result, this study provides valuable insights, for technology providers of smart products for livestock farming, as well as for policymakers.

**Abstract:**

In recent years, the fourth industrial revolution has found its way into agriculture. Under the term smart farming, various so-called smart products are offered that may positively influence both the daily work of farmers and animal welfare. These smart products can collect data from the farm, extract important information, and in some cases even make decisions independently. Particularly in Germany, where intensive livestock farming is criticized by society, such smart products could make a significant contribution to improving animal welfare. However, an important prerequisite is the acceptance of the users, who are usually the livestock farmers themselves. So far, there is little knowledge about farmers’ attitudes towards smart products in livestock production. In this study, a factor analysis and a cluster analysis are conducted to evaluate the attitudes of German livestock farmers towards smart products. Based on the analysis of an online questionnaire in which German livestock farmers (n = 422) participated, four clusters could be derived. The main distinguishing characteristics of the clusters are the influence of the social environment, the expected effort for implementation, the general trust in smart products, and the technology readiness of the farms. As a result, this study provides valuable insights for technology providers of smart products for livestock farming as well as for policy makers.

## 1. Introduction

Intensive, conventional livestock farming has been the subject of vehement public criticism for several years. In addition to reasons related to the way in which the animals are kept themselves, such as the space provided per animal, animal welfare issues, animal–human interaction, and overarching scandals in food supply chains are further responsible for the fact that the professional image of farmers and livestock farmers is deteriorating [1]. In addition to animal welfare, livestock farming and agriculture further play a key role within the international debate on climate change and sustainability, especially with regard to the sustainable use of natural resources [2]. As a consequence, a strong aversion to current systems of intensive livestock farming prevails, specifically for pigs and poultry, but also in dairy farming [3]. Furthermore, the sensitivity towards agriculture along with requirements aimed at improving farm animal welfare standards is increased [4,5,6,7]. Common choices for livestock producers to counter criticism include open farm tours for everyone or live streaming from livestock facilities. Another way for farmers to respond to societal and policy demands and provide a higher standard of farm animal welfare is to participate in specialized animal welfare programs [8,9]. For instance, one such animal welfare program was established in Germany in 2015 under the name of the Initiative Animal Welfare (IAW). Its objective is to improve farm animal welfare in German poultry and pig production. Based on current consumer surveys, it can be inferred that the IAW program is very well accepted overall by consumers in Germany [10]. Since 2020, a total of 4200 pig farmers (24.7% of total pig farmers in Germany), and 2000 chicken farmers (60% of total meat chicken farmers in Germany) have participated in the IAW [11]. However, recent studies demonstrate that farmers perceive a great effort with participation in animal welfare programs, which significantly diminishes their intention to participate [12,13,14]. Since Germany represents one of the largest meat producers worldwide, it is considered to act as one of the main players on the international market [15]. For this reason, it can be assumed that examples and results from Germany can also be relevant on an international level. Thus, the question arises how to facilitate farmers’ work and especially the effort associated with livestock farming in a profitable way.

In the last few years, the fourth industrial revolution has found its way into agriculture, driven by the continuously increasing diffusion of information and communication technologies. Smart farming is expected to enable the development of a more sustainable agriculture and livestock farming through its technologies [16,17]. In the field of livestock farming, smart ear tags for dairy cows were developed, which enable monitoring of individual animal behavior by tracking the movements of cows within the stable environment in real time [18]. Furthermore, lameness detectors are used that can automatically detect lameness in cows in real time and inform the farmer [19]. Smart ear tags equipped with accelerometers are further applied in sow farming for early lameness detection [20]. Common features of these smart livestock technologies include inherent supportive reconfiguration capabilities to perform agile actions in real time, especially in the event of sudden changes in farm operating conditions [21]. In addition, smart products are often referred to as intelligent devices or products that are interconnected and interact via local and global, often wireless, network infrastructures, thus serving as a link between the virtual and physical world by responding in real time and autonomously making decisions [22]. Smart products distinguish themselves from classical cyber-physical systems, e.g., by haptic user interfaces based on gesture identification or acceleration sensors. They are characterized by using specific technologies to capture and communicate information about themselves, their condition, and the surrounding environment [23]. The benefits of smart livestock farming are considered highly important, for example, by facilitating work time or simplifying animal monitoring. Regarding climate change and sustainability, it is assumed that smart farming reduces the ecological footprint of agriculture and mitigates greenhouse gas emissions in livestock farming as well as livestock monitoring and disease detection [24]. Nevertheless, in addition to technological and organizational challenges, hurdles remain in the adoption of these technologies. These include, for example, data security and data sovereignty, high costs of implementation, or limited farmer skills and knowledge [25,26].

A fundamental condition for benefiting from the advantages of smart products in terms of farm animal welfare is farmer acceptance and use [27]. Previous studies have concentrated incrementally on technologies in the context of precision farming, while adoption of smart livestock farming has rather been neglected. For example, the relevance of the role of farm attributes and socio-demographic characteristics of farmers, such as education or age have been investigated [28,29]. With regard to smart products, only the use and acceptance of smartphones among farmers has been studied in Germany to date [30]. Further empirical studies explicitly investigating the acceptance of smart products in German livestock farming are non-existent. However, it is known that livestock farmers cannot be considered as a homogeneous group in terms of their attitudes and that a positive attitude also has an effect on acceptance as well as actual behavior [31]. For this reason, and to address the research gap, livestock farmers in Germany were surveyed about their attitudes and (potential) usage behavior with regard to smart products. A factor analysis and subsequently a cluster analysis was approached for this article in order to identify potential target groups for the use of smart products and to characterize them in more detail so that target group-specific managerial implications can be derived. The results bear important implications for manufacturers and policymakers at national and international levels, resulting in opportunities to tailor smart products to the needs and wishes of livestock farmers.

## 2. Materials and Methods

### 2.1. Study Design

A questionnaire among livestock farmers in Germany from June to August 2020 was analyzed for this article. It was based on an anonymous and standardized online questionnaire. The questionnaire was pre-tested for one week by different experts from agro-economic research and farmers. To reach as many livestock farmers as possible, and to achieve as much homogeneity, especially with regard to personal characteristics, compared to the basic population of livestock farmers in Germany, it was then distributed via a chair’s own mailing list and via the Lower Saxony Chamber of Agriculture. Private networks and social media, such as Facebook, Xing, and Instagram, were used to further distribute the link to the questionnaire. Further magazines in agricultural practice were asked to distribute the questionnaire (topagrar, profi, farm food future, Oldenburger intenational, der Hoftierazt). The questionnaire was preceded by a brief description of the background and objectives of the study, namely the investigation of livestock farmers’ attitudes towards the use of smart products. Furthermore, a definition of smart products was prefixed to the questionnaire (“More and more devices are connected to the internet. Smart products collect data via corresponding sensors, analyze them and forward them via the internet or receive data from other smart products. The “intelligence” of these products means that they perform tasks independently and communicate with other products”). Three examples were given: smart phone, smart ear tag, smart pig counter. Based on the extended “unified theory of acceptance and use of technology” model, the questionnaire was constructed [32]. With its key constructs being performance expectancy, effort expectancy, social influence, facilitating conditions, hedonic motivation, price value, and habit, it provides a conceptual framework for analyzing decision makers’ intention to use or not to use a certain technology or system. Further, it has been applied to investigate acceptance issues for technologies or systems within agriculture [33].

The questionnaire mainly consisted of closed questions. It was also divided into three parts. Part A asked farmers about socio-demographic and farm characteristics, such as age, gender, education, work experience, farm size, and individual farm operations. Part B consisted of 55 statements designed to determine farmers’ attitudes toward smart products. The respondents were asked to rate predefined statements on the basis of five-point Likert scales from 1, meaning, “I totally disagree” to 5, meaning, “I totally agree”. Part C consisted of 10 statements designed to determine farmers’ perceptions of national adoption of smart products. 

### 2.2. Statistical Analysis

IBM SPSS Statistics 26 software for Microsoft was utilized to perform the factor and cluster analyzes as well as the descriptive analysis of the questionnaire. First, the data were cleaned regarding incompleteness and non-livestock farmers as well as outliers. In order to obtain an overview of the sample, socio-demographic and farm characteristics were analyzed using univariate methods. Subsequently, an exploratory factor analysis was conducted to combine a large number of items into a smaller number of factors, allowing for a simplified interpretation of farmers’ attitudes towards the use of smart products. A principal component analysis was applied for this article using the varimax rotation method and Kaiser Normalization. The varimax rotation was exerted because the variance of the squared loadings reaches the maximum value with this rotation and, thus, facilitates the interpretation, as the assignment of statements to the respective factors is simplified [34]. Despite the variables of the Unified Theory of Acceptance and Use of Technology, Likert-scaled items on farmers’ trust and technology readiness with respect to behavioral intention and use of smart products on their farms were included in the factor analysis. The before mentioned number of items was tested for suitability for factor analysis. The Kaiser–Meyer–Olkin (KMO) criteria and the “measure of sampling adequacy” (MSA) were applied for this purpose. If KMO and MSA are below 0.5, the result is considered “unacceptable” and factor analysis should not be performed [35]. In this study, a value of 0.930 was determined, which is classified as “marvelous” [36]. Another quality criterion is Bartlett’s test of sphericity, which assesses the null hypothesis so that the input variables from the survey population are in uncorrelated form [36]. In this study, Bartlett’s significance is at a value of *p* = 0.000, which is highly significant because it means that the null hypothesis can be rejected and thus there is a correlation between the variables. Moreover, as in principal component analysis, only those factors that have an eigenvalue greater than 1 are extracted. Here, the factor analysis explained 59.03 percent of the total variance. Finally, seven factors including 34 items were identified (see Section 3.2). A reliability analysis provided information about the internal consistency of the factors. Cronbach’s Alpha value was chosen to measure the consistency. All seven factors show a valid internal consistency [37,38].

Afterwards a hierarchical cluster analysis was performed to classify farmers into different segments in terms of their attitudes towards smart products. For this purpose, the previously formed factors were applied to characterize the clusters. This type of cluster analysis is utilized to generate homogeneous groups from a heterogeneous population [36,39]. The process of conducting the cluster analysis was organized into three steps. Initially, the single-linkage method was performed to remove eventual outliers. The objects with the smallest linkages were combined with another. Then, the optimal number of clusters was determined based on Ward’s method. The analysis was successful in identifying four clusters. The dendrogram, a screen plot, and the elbow criterion were adopted to support the decision on the optimal number of clusters [36]. Eventually, a k-means clustering was performed to optimize the Ward’s results. Discriminant analysis was applied to verify the results. In this study, 100.00% of the original cases were correctly classified [36,38].

Additional results of the discriminant analysis (eigenvalues and Wilks–Lambda) further prove that the results of the cluster analysis are of high quality. Wilks–Lambda is a significance test that confirms a significant difference between the groups identified in this study. In addition, the eigenvalue was greater than 1, and significant differences (*p* < 0.000) were found between the clustering factors [37]. No significant difference was found between the clusters with regard to the factor Performance, which was therefore not included in the cluster analysis. For a detailed characterization of the generated clusters, the Tamhane T2 post-hoc multiple comparison test was conducted, which demonstrated significant differences between the clusters. The Tamhane T2 test is based on the assumption that the groups may differ in variance. For a more detailed description and to test significant differences between the clusters, a univariate ANOVA, and a cross-tabulation were applied [38]. In addition, the clusters were analyzed with respect to socio-demographic and farm characteristics.

## 3. Results

### 3.1. Sample Description

A total of 422 complete data sets were available for analysis. Moreover, 24.4% of the respondents were female. Since the proportion of female employees in German agriculture is about 36%, women are slightly underrepresented in this study [40]. The majority of respondents live in Lower Saxony (22.3%), North Rhine-Westphalia (20.1%), and Bavaria (14.7%). The same federal states have the highest share of professional farms in total farms in Germany [41]. Moreover, 20.9% of the respondents are older than 55, slightly underrepresenting the average age of employees, as one-third of all agricultural employees in Germany are older than 55 [41]. The average farm size is 375.15 hectares, which is significantly higher than the average number of hectares per farm (61.9 hectares) in Germany [42]. Furthermore, 37.9% of the respondents hold an agricultural apprenticeship, have attended a special agricultural college, or have a Meister (master farmer) degree. In this case, the average of the educational distribution of German farmers, where 68% have the above types of educational qualifications, is fallen rather short [40]. Moreover, 42.9% of the respondents in the sample hold a university degree. Overall, the proportion of farmers with a university degree in Germany amounts to 12%, which leads to an overrepresentation of academics [40]. Furthermore, 65.8% of the respondents stated that they already use smart products (including smart phones) on their farm. Due to the small sample size and the sampling procedure (network sampling), this sample cannot be considered representative for all German livestock farmers with regard to the participants’ farm characteristics. Nevertheless, due to the highly significant results, these may provide interesting insights, especially for large full-time livestock farms.

### 3.2. Results of the Factor Analysis

The final factor solution included seven factors with 34 statements (see Table 1). The means (μ) and standard deviations (σ) are also plotted in Table 1. The first factor, “performance”, describes the farmers’ assessment of the operational benefits of using smart products and contains ten statements (Cronbach’s alpha: 0.891). Overall, the factor “performance” is assessed positively. The second factor, “animal benefits”, covers farmers’ attitudes with respect to livestock, and contains four statements (Cronbach’s alpha: 0.863). It is rated positively in general. The third factor, “social environment”, describes the influence that the social surrounding exerts on farmers in connection with smart products (Cronbach’s alpha: 0.779). The factor “social environment” varies and displays an almost indifferent picture on average. The fourth factor, “effort”, summarizes four statements about the effort incurred by using smart products (Cronbach’s alpha: 0.770). On the overall average, the effort is rated low. The fifth factor, “trust”, summarizes five statements about farmers’ attitudes toward trusting smart products (Cronbach’s alpha: 0.744). The overall average presents an indifferent picture. The sixth factor, “technology readiness”, combines four statements about the systemic conditions that should be fulfilled for the adoption of smart products (Cronbach’s alpha: 0.652). Although the overall average is positive, high standard deviations are evident. The seventh factor, “facilitating conditions”, describes the basic framework conditions that simplify the use of smart products on the farm (Cronbach’s Alpha: 0.728). Similar to technology readiness, high standard deviations are also observed for the factor “facilitating conditions”. 

The performed tests to check the quality of the factor analysis indicated that all factors met the common requirements. The factor analysis explained 59.03 percent of the total variance among the 34 statements. Since the KMO value is relatively high at 0.930, these statements are well suited for cluster analysis. The identified factors were adopted as clustering variables to form groups for smart products attitudes.

### 3.3. Results of the Cluster Analysis

Based on the factors identified, a cluster analysis was conducted. The aim of the cluster analysis was to divide the farmers into groups according to their attitudes toward smart products. The factor performance had to be eliminated from the cluster analysis because no significant differences between the groups were identified. Table 2 presents the results of the cluster analysis, the mean values of the cluster-forming factors and the underlying variables. 

The first cluster (cluster A) contains 96 livestock farmers. It is characterized by an overall positive attitude towards the use of smart products and acts independently of its social environment, therefore entitled “independent proponents”. On the one hand, they are of the opinion that animal pathologies could be detected faster with the help of smart products (μ = 4.19; σ = 0.812) and, on the other hand, that they would let smart products assist them in animal control (μ = 4.32; σ = 0.900). Moreover, cluster A is independent of its social environment, especially in terms of society’s expectations (μ = 2.25; σ = 0.795) and colleagues’ opinions (μ = 2.63; σ = 0.861). Farmers tended to reject the statements that safe handling (μ = 2.77; σ = 1.192) and learning to handle (μ = −2.36; σ = 1.027) smart products was difficult. Cluster A farmers show little concern regarding statements related to trust. They would follow a recommendation that a smart product provides them (μ = 3.72; σ = 0.706) and demonstrate a basic trust in technologies and machines (μ = 3.92; σ = 0.816). In addition, they exhibit a high level of technology readiness, both because they use various sensors on their farm (μ = 4.84; σ = 0.443) and because computers are deployed for specific tasks, such as herd management (μ = 3.96; σ = 1.123). The statement indicating that internet access or mobile connection is available on the entire farm tends to be agreed with (μ = 3.51; σ = 1.384). Compared to clusters B, C, and D, farmers in cluster A show the least influence by their social environment. At the same time, they show the highest trust and the highest technology readiness given not entirely optimal internet conditions on their farm. The results of the cluster analysis reveal relatively high standard deviations within certain statements. This indicates the existence of heterogeneous attitudes within the individual groups, especially with regard to their technological readiness and the facilitating conditions.

The second cluster (cluster B) comprises 65 livestock farmers. It is characterized by a high perception of the benefits of smart products, while the technology readiness and facilitating conditions remain insufficient. Farmers in this cluster are declared as the “hindered adopters”. Their opinions of possible benefits in livestock management are highly positive when it comes to obtaining real-time information about the animals’ condition (μ = 4.32; σ = 0.900) or that animal pathologies can be detected more rapidly (μ = 4.31; σ = 0.789). In addition, cluster B assumes that its social environment exerts little influence on its attitude toward smart products, especially regarding colleagues (μ = 3.20; σ = 1.003) and society’s expectations (μ = 2.62; σ = 1.041). In terms of effort related to the use of smart products, farmers in cluster B show no expected difficulties. This mainly concerns the learning effort (μ = 2.02; σ = 0.992) as well as the potential stress caused by the use (μ = 1.97; σ = 0.918). In addition, farmers in cluster B trust smart products in general. They are rather indifferent whether their data are secure when they work with products from reputable manufacturers (μ = 3.38; σ = 1.071), but assume that they receive sufficient utilization-related information from the manufacturer (μ = 4.00; σ = 0.866) in case of purchase. In terms of technology readiness, cluster B does not perform particularly well. They tend to negate statements that computers are involved in various tasks on the farm like herd management (μ = 2.03; σ = 1.104) and that information systems on the farm are capable of autonomous decision processes (μ = 1.47; σ = 0.730). In general, farmers from cluster B continue to rather lack facilitating conditions as the availability of a stationary or mobile internet access on the entire farm (μ = 2.68; σ = 1.147). Compared to clusters A, C, and D, cluster B considers smart products to provide the highest benefit toward livestock and the lowest effort in learning costs and handling, while they tend to barely meet the technically necessary prerequisites for adoption.

The 157 farmers in the third cluster (cluster C) qualify as “unrestrained promoters” since they intend to adopt smart products in principle and fulfill the technical and operational requirements at the same time. Farmers in cluster C indicate that the use of smart products offers great advantages, as abnormalities in the animals’ natural behavior can be detected more rapidly (μ = 4.38; σ = 0.583). They would further unquestionably let a smart product assist them in animal control (μ = 4.47; σ = 0.656). Regarding the statements about the role of the social environment, it becomes apparent that especially the family (μ = 4.20; σ = 0.755), as well as society (μ = 3.86; σ = 0.763) markedly influence the farmers’ attitude. They do not perceive a great effort in learning how to use smart products (μ = 2.38; σ = 0.1.034) and do not consider the use as a stressor (μ = 1.97; σ = 1.032). Regarding trust, farmers of cluster C appear indifferent up to positive. They tend to agree with the statement that they trust smart products and their decisions (μ = 3.56; σ = 0.737) and would tend to follow a recommendation received from a smart product (μ = 3.66; σ = 0.694). Overall, they show very favorable farm conditions, which is confirmed by statements concerning the presence of an internet connection on the entire farm (μ = 4.15; σ = 1.051). Compared to clusters A, B, and D, they are most affected by the social environment, whereas at the same time they feature the most favorable digital environment in terms of Internet access.

A total of 104 farmers were assigned to cluster D. Since the farmers in this cluster are highly skeptical, they are referred to as the “trustless critics”. Cluster D is mostly indifferent to the benefits a smart product could confer on livestock. This is reflected in the statements about whether animal diseases can be detected more rapidly (μ = 3.07; σ = 1.026) and whether farmers would let a smart product assist them in animal control (μ = 2.92; σ = 1.129). With regard to the social environment, cluster D is indifferent or independent. This is evident both in statements of a potential positive impression in society (μ = 3.12; σ = 0.938) and in the context of endorsement within the family (μ = 3.09; σ = 0.946). In addition, cluster D expects a slightly increased effort in the adoption of smart products, on the one hand with regard to the operation (μ = 3.28; σ = 1.038), on the other hand with regard to the stress caused by utilization (μ = 3.10; σ = 1.084). Trust in smart products tends to reveal a critical image. Questions about trust in smart products and their decisions (μ = 2.63; σ = 0.926) and about data security when working with smart products from reputable manufacturers (μ = 2.48; σ = 0.985) are answered doubtfully. Although various sensors are applied on farms from cluster D (μ = 4.11; σ = 0.944), autonomous decisions are not performed by information technologies (μ = 1.71; σ = 0.889). Hence, technology readiness tends to be limited. Furthermore, internet access for farmers from cluster D is limited on the farm (μ = 2.76; σ = 1.235), which constrains the conditions for smart product adoption. Compared to clusters A, B, and C, farmers in cluster D assess the animal benefits from smart products the lowest. At the same time, they evaluate the effort the highest and express the greatest concerns about trust. However, as with cluster A, high standard deviations occur, which indicate partially heterogeneous attitudes within the cluster.

Table 3 shows some characteristics within the analyzed clusters. Most of these differences are at significant levels. Compared to the others, cluster B contains the highest proportion of women. In terms of age, it is also apparent that farmers from cluster B are on average about eight years younger. At the same time, farmers from cluster B hold on average the least amount of hectares of land, significantly less than cluster A and C. Furthermore, it becomes apparent that cluster B includes most of the farmers in a secondary occupation. Furthermore, farmers from cluster B average nine years less work experience. Few significant differences between clusters were identified with respect to the type of livestock farming. These were evident in sow keeping, dairy cattle, and horses. Thus, the fewest sow keepers are located in cluster B. Cluster D contains the smallest number of dairy farms, while the proportion in clusters A and C is rather large. Furthermore, the share of horse keepers is highest in cluster B. Beyond socio-demographic and farm characteristics, significant differences were discovered in behavioral intentions regarding the use of smart products. On the question of whether the intention to use smart products in the near future is present, clusters A and C show significant affirmation, whereas cluster D tends to negate this intention. Eventually, significant differences were revealed in the actual use of smart products. In particular, clusters A and C show a high proportion of farmers who already use smart products on their farms. In contrast, the proportion in clusters B and D is significantly lower. Usage was further assessed in terms of frequency of use. Initially, it emerged that clusters A and C most frequently use their smartphones for non-communicative purposes compared with clusters B and D, for example to monitor or control machinery or livestock facilities. Small machines or devices that perform tasks independently without human intervention are used less frequently, though least in clusters B and D.

## 4. Discussion

This study analyzed data from 422 livestock farmers who participated in an online questionnaire about their attitudes toward smart products. By applying a cluster analysis with a factor analysis in advance, four clusters of farmers could be formed. Cluster A (“independent proponents”) is characterized by its support for smart products. It shows the least influence of the social environment and at the same time the highest level of trust and technology readiness. Cluster B (“hindered adopters”) demonstrates a strong intention to use smart products. It evaluates the benefits in terms of livestock the highest and rates the effort as very low. However, it is the weakest in terms of technology readiness and facilitating conditions. Cluster C (“unrestrained promoters”) likewise shows a distinctly positive attitude toward smart products. It is most strongly influenced by the social environment. At the same time, it is most likely to feature facilitating conditions and similarly exhibits a high level of technology readiness. Cluster D (“trustless critics”) displays the strongest distrust of smart products. The farmers in this cluster perceive only a minor benefit for the livestock, consider an increased effort to learn operation and handling, and lack trust in smart technologies.

Performance is defined as the extent to which a person believes that using the system or a technology, in this case smart products, provides a benefit. Based on the statements of the factor analysis, performance in relation to the use of smart products consists of a higher profit as well as the possibility to reduce working hours and to facilitate and accelerate work processes. In addition, performance includes the ability of farmers to produce more sustainably with the help of digitization. Although no significant differences were found among the four clusters, performance expectancy plays a role in the adoption of new technologies and systems. Economic aspects are considered as one of the main reasons for farmers to adopt smart farming technologies [21]. Expected performance in terms of profit further favors farmer adoption of a technology due to the prospect of more stabilized revenues [43]. In addition, expected financial performance represents an important positive influence on the adoption of sustainable practices in agriculture [44]. Furthermore, the opportunity itself to contribute to a more sustainable agriculture is perceived as an advantage of smart products in the overarching context of smart farming [16]. However, the ability to facilitate the control and management of livestock is another perceived benefit [45,46]. Even though performance was identified as a factor, it could not be included in the cluster analysis because no significant differences between the groups could be observed. Therefore, all clusters perceive certain benefits from the use of smart products.

Animal benefits are defined as the degree of expectation an individual believes that the use of a system or technology, in this case smart products, will be beneficial and provide support and relative benefits to the livestock. Many farmers consider the constant monitoring and detection of changes in natural behavior as well as the early identification of diseases as a benefit of smart livestock farming [47]. Regarding the improvement of farm animal welfare, personal motivation, and joy in improving farm animal welfare is considered an important motivator, although it is often suppressed by financial incentives [48]. However, the care and responsibility for the livestock as well as the maintenance of animal health also contribute to the personal satisfaction [49]. Therefore, it can be assumed that these factors may also affect the farmers’ use behavior of smart products. The individual clusters differ significantly in their attitudes toward animal benefits. While clusters A, B, and C anticipate distinct benefits for livestock from the use of smart products, cluster D is considerably more skeptical, and would tend not to let a smart product assist them in monitoring livestock.

Social influence is determined by important people, such as friends, colleagues, and relatives, who influence the individual to use a system or technology. Further, the role of society is also considered. Social pressure from society in particular exerts discomfort on livestock farmers, which was revealed in a study on the willingness to participate in animal welfare programs [50]. Research that examined the social influence of colleagues, friends, and family on strategic decisions on the farm identified a contribution of social influence related to issues such as sustainable agriculture and environmental conservation [51]. In addition, the experience of a farmer’s colleagues with new technologies, such as smart products was determined to significantly influence the farmer’s future use [52]. Moreover, family members greatly affect strategic decisions regarding the development of the farm [53]. Adopting smart products also represents a strategic decision for a farm. For farmers, familiar social contacts and interaction with colleagues are particularly important for successful technology implementation, as their learning processes are primarily socially oriented [54]. The effect of social influence is most evident in cluster C. Farmers from cluster A in particular appear to be independent of social influence. The influence of the social environment can also be observed in clusters B and D, albeit to a lesser extent.

Effort in this article refers to the expected and actual effort required to implement and use smart products. Previous studies on animal welfare programs suggest that the additional workload associated with participation is criticized by many farmers. This particularly applies to the temporal burden due to documentation duties [55]. A different study found that willingness to participate in animal welfare programs decreases as implementation efforts increase [56]. Studies on precision agriculture report that farmers face adaptations in their farm by shifting from experience-based decisions to data-driven processes, leading to uncertainties about the potential costs of the technology [26,57]. At the same time, the demands on farmers’ knowledge and skills increased due to the growing complexity of technologies [47]. Farmers’ experiences with previous technology implementations, where production increases and labor savings were overestimated, may also contribute to unmet expectations and critical attitudes toward innovative technologies [57]. Therefore, here, the above-mentioned thesis that the willingness to use smart products decreases with increasing effort can be agreed to. Farmers in clusters A, B, and C do not appear to perceive learning how to use smart products as an actual effort at all, compared to cluster D. The same is evident in the questions about the perception of handling simplicity and a possible stressor that could emanate from smart products.

Trust is not only an essential factor for a successful business relationship in the agricultural sector, but also substantial for the adoption of new technologies. Regarding the implementation of farm animal welfare-oriented measures, the extent to which farmers consider the implementation of a certain measure as important and reasonable in order to ensure sustainable livestock farming appears to play an overarching role [58]. Further, trust appears to be an impactful factor in the adoption of technologies, such as smart products. Empirical research for example suggests that low rates of technology adoption are more often caused by lack of confidence than by cost. Even if farmers discover the potential benefits of the technology, some lack confidence that the new technology will work as purported [59]. Simultaneously, increasing confidence by believing that a technology will deliver the expected results, as well as comprehending the process to achieve an objective with a technology, is assumed to lead to higher adoption rates of a technology [60]. Similar results emerged in the study of farmer adoption of innovative green technologies [61]. Particularly, at this point, the present results highlight that the population of farmers cannot be understood as a homogeneous group. Four clusters of farmers were found that differ in their attitudes and willingness to use smart products (Table 2). These results indicate that livestock farmers in clusters A, B, and C would tend to follow a recommendation that a smart product provides them, while cluster D rejects the statement. When asked about data security, clusters B and C show no trust-related concerns. Cluster A is overall indifferent regarding this statement whereas cluster D is characterized by massive distrust.

Technology readiness refers to the availability of sufficient resources and organizational knowledge to successfully implement smart products [62,63]. Technology readiness thus encompasses the extent to which an organization is prepared for a technology implementation. It includes general information technology systems such as internal networks and specific information technology systems required to support the system [64]. Another factor is the extent to which organizational operational processes are aligned with the implementation challenges and can be adapted to the new system requirements [65]. In addition, it includes the extent to which an organization possesses or is able to acquire the necessary technical skills, knowledge, and workforce to implement a technology [66]. One of the few studies that investigated technology readiness for smart farming technologies on farms also emphasized the importance of technology readiness for adoption [67]. The clusters distinctly vary in their technology readiness. On the farms of clusters A and C, for example, computers and sensors are regularly utilized. Farmers from cluster D may use sensors, but computers are seldom utilized. The question of whether information technology systems independently make decisions on the farm was rather negated overall. This is least the case for clusters B and D. 

The construct facilitating conditions describes to which degree the respondents believe that a favorable infrastructure exists on the farm that facilitates the use of the system. In this study, facilitating conditions refer primarily to broadband coverage and the availability of an Internet connection. If farmers believe that a system or technology matches their needs and is compatible with their environment, it is considered likely that the technology will be adopted because they consider it a positive investment [68]. Furthermore, the availability of the necessary infrastructure for a technology such as smart products also represents facilitating conditions [21]. A previous study also indicated that a favorable farm environment increases the likelihood that a farmer will adopt a technology [69]. Cluster C exhibits the best prerequisites in terms of facilitating conditions with regard to Internet accessibility. Cluster A displays a similar tendency. However, it is noticeable that cluster D possesses limited Internet conditions, while cluster B ranks the weakest.

From the above-mentioned factors, several opportunities emerge to enhance the attractiveness of smart products use by livestock farmers. These recommended approaches address manufacturers that develop and offer smart technologies, and policymakers in particular. Farmers from cluster A (“independent proponents”) are unconditionally positive towards smart products and independent from their social environment. This is also reflected in their behavioral intention and actual use behavior. Around four out of five farmers from cluster A already use smart products in their livestock and intend to continue in the future. Recommendations for action in cluster A address in particular policymakers, who should continue to improve broadband coverage in Germany. Farmers from cluster B (“hindered adopters”) tend to express highly positive attitudes towards the benefits of smart products and perceive their usage as effortless, while they rather lack technology readiness and facilitating conditions. It is assumed that for these reasons uncertainty about the intention of use prevails, as it complicates the use. Furthermore, it is remarkable that the livestock farmers in cluster B are significantly younger and consequently exhibit less work experience in terms of time than in the other clusters. In addition, cluster B contains more women and the amount of farmland is smaller than it is in the other clusters. Recommendations for action, similar to cluster A, are primarily directed at policymakers. This includes the promotion of nationwide broadband coverage, since the majority of agriculture is practiced in rural areas. Similar to animal welfare programs, policy makers could consider establishing financial incentives to promote smart livestock farming technologies that are proven to improve animal welfare in livestock farming. This idea is supported by the fact that livestock farmers perceive a distinct benefit for their livestock, but are stymied by the technical infrastructure required. Livestock farmers from cluster C (“unrestrained promoters”) strongly support smart products, underlie the influence of their social environment, and fulfill all prerequisites regarding technology readiness and facilitating conditions for the adoption of smart products. The use behavior clearly reflects this. With regard to socio-demographic and farm characteristics, it is evident that livestock farmers from cluster C operate the largest farms regarding farm size. The only recommendation for action for manufacturers and policymakers at this point is to continue to support these livestock farmers. 

Livestock farmers from cluster D (“trustless critics”) represent the most critical of smart products by far. They perceive no specific benefit to livestock, find increased effort to learn and use smart products, and lack trust in them. This is further reflected in the behavioral intention and use behavior. However, the only notable finding in the farm characteristics is the significantly lower number of dairy farmers than in clusters A and C. At first, it may be stated that compared with cluster A, B, and C, a substantially higher effort is required to motivate farmers of cluster D to use smart products. Recommended approaches focus especially on manufacturers and policymakers. Given the lack of obvious benefits to livestock farmers regarding their livestock, manufacturers should communicate the benefits and limitations of smart products simply and clearly. For instance, as additional documentation requirements are perceived as a heavy burden by farmers when participating in animal welfare programs, manufacturers should highlight benefits of smart products in facilitating documentation requirements or livestock monitoring. Since the expected effort also exerts a major influence on attitudes toward the use of smart products, usage should be associated with a positive experience, which is a reason for designing products that are simple and user-friendly in terms of haptics and user interface in order to facilitate uncomplicated use. This likewise includes ensuring that learning how to use and handle smart products becomes as easy as possible in order to reduce uncertainty and complexity. Hence, trust strongly influences the behavioral intentions of livestock farmers from cluster D, the exchange of monitoring data is an important aspect that can be achieved, for example, by establishing internationally binding legal standards. Manufacturers should further focus on service and, in the case of livestock farmers, especially on availability. Proper installation, necessary maintenance, and support for operational and problem issues form part of this. Another way to achieve greater trust can be to directly involve farmers in the development of smart products. This involves open communication with farmers and actively finding compromises. As facilitating conditions are one of the most important determinants for the use of smart products by farmers, the government should provide the necessary support for adoption, including the development of a more widespread broadband coverage. Further, the government could consider providing monetary incentives for the implementation of smart products, insofar as they contribute to enhancing farm animal welfare standards. To increase farmers’ trust, policymakers should remove ambiguities around data security and data sovereignty. Like most non-experimental studies, this study has some limitations that should be considered when interpreting the results. First, the study provides non-representative results due to the modest sample size of 422 farmers and the different composition of the sample compared to the population of German livestock farmers. The farmers in this study operate farms with an average of 375.15 hectares, which is markedly higher than the average for German farmers [42]. Nevertheless, due to the highly significant results and Germany’s role at the world market, these may provide interesting insights, especially for large full-time livestock farms. In addition, conducting an online survey certainly entails certain limitations. While it is easy to implement and realize, it may limit representativeness due to differences in internet use in certain segments of the population. Women, elderly persons, and persons with low education use the internet less frequently than men, younger persons, and persons with higher education [70]. Conducting the survey online enables some segments of the population to be reached more easily than others. Individuals who describe themselves as technology-averse might choose not to participate in an online survey in the first place. In addition, no response rate can be determined from the online survey, as no information is available on how many people received the link via the various mailing lists. An additional point of discussion is the occurrence of occasional high standard deviations within the results, which indicates that the attitudes of livestock farmers still differ within a cluster. This applies, in particular, to cluster D. Finally, it is worth mentioning the subjective nomenclature of the clusters, which originated from the authors, and therefore should not be considered as generally binding.

## 5. Conclusions

The aim of this article was to identify groups of livestock farmers that differ in their attitudes toward smart products and to analyze these groups. This objective was achieved by grouping livestock farmers into four clusters that differ significantly. These clusters further demonstrate differences between their socio-demographic and farm characteristics at a significant level. Based on the results, it is possible to deduce what is relevant for the farmers in each cluster. Important findings of this article include that technology readiness, trust, and the social environment exert a substantial influence on farmers’ attitudes towards smart products. At the same time, it becomes evident that certain performance expectancy is prevalent among all farmers surveyed, which is the reason why performance expectancy is not suitable as a delimiting criterion between the clusters.

Future research could focus more precisely on the individual animal types and livestock housing systems. In addition, it could be scientifically investigated how the use of smart products actually affects the working time spent. One possible research method to draw comparisons regarding the temporal and financial situations of the livestock farmers could involve work diaries. These results could provide more detailed information than those obtained in the present study and would therefore deliver very interesting managerial results. Furthermore, the economic effects of using smart products in livestock farming could be analyzed by comparing efficiency aspects such as profitability, liquidity, and stability between farms using smart products and those not using smart products with the help of matching algorithms. In addition, farmers’ opinions should be taken into account for the future and compared separately between users and non-users of smart products. For this purpose, choice-based experiments could serve to explore under which conditions farmers would implement specific products. In addition, it would be interesting to investigate how the use of smart products actually affects animal welfare.

## Figures and Tables

**Table 1:** Results of the factor analysis.

Factor and Statements	Factor Load	μ	σ
Factor 1: Performance (Cronbach’s Alpha: 0.891)			
Using smart products can reduce the working time for certain activities.	0.721	4.09	0.873
By using smart products, labor costs can be reduced.	0.719	3.68	0.942
Smart products can relieve me of certain work processes.	0.694	4.17	0.812
By using smart products, I save time on routine tasks on my farm.	0.686	3.83	0.970
Smart products can accelerate work processes (e.g., communication, decisions).	0.655	4.09	0.875
With smart products, I can operate more efficiently.	0.581	3.79	0.928
Using smart products quickly becomes a habit.	0.525	4.13	0.780
By using smart products, a higher profit can be generated.	0.509	3.26	0.900
Using smart products results in lower costs because mistakes due to human failure are minimized.	0.487	3.34	0.923
I want to produce more sustainably by making the best use of resources with the help of digitization.	0.482	4.12	0.935
Factor 2: Animal benefits (Cronbach’s Alpha: 0.863)			
Animals can benefit from smart products because deviations from normal behavior are detected more quickly.	0.779	4.04	0.928
Animals can benefit from smart products because diseases are detected more quickly.	0.762	3.96	0.979
I would have a smart product assist me with animal control.	0.738	4.03	1.078
I am pleased when smart products can inform me about the condition of my animals in real time.	0.683	4.19	1.019
Factor 3: Social environment (Cronbach’s Alpha: 0.779)			
I think using smart products makes a good impression in society.	0.777	3.30	1.016
I think society expects me to use smart products.	0.697	2.82	1.078
My social environment (neighbors, colleagues, friends), approves of smart products on my farm.	0.684	3.27	1.073
I think that my colleagues will like it if I use smart products on my farm.	0.625	3.28	1.018
My family is in favor of smart products on my farm.	0.419	3.65	1.006
Factor 4: Effort (Cronbach’s Alpha: 0.770)			
Learning how to use smart products is difficult for me.	0.823	2.43	1.091
Safe handling of smart products is not easy for me.	0.741	2.68	1.179
I imagine the operation of smart products to be difficult.	0.712	2.79	1.003
Using smart products is a stressor for me.	0.657	2.44	1.103
Factor 5: Trust (Cronbach’s Alpha: 0.744)			
I am confident that machines work the way they are programmed.	0.704	3.62	0.885
I trust smart products and the decisions they make.	0.645	3.28	0.905
In case of purchasing new smart products, I will get enough information from the manufacturer to use them reasonably.	0.611	3.57	0.981
I think my data are safe when I work with smart products from reputable manufacturers.	0.488	3.09	1.089
I would follow a recommendation that a smart product gives me.	0.442	3.50	0.767
Factor 6: Technology readiness (Cronbach’s Alpha: 0.652)			
Various sensors are used on my farm.	0.744	3.41	1.318
On my farm, computers are used for certain tasks (e.g., herd management).	0.740	4.35	1.011
I am used to adapting the farm to changing conditions.	0.520	4.22	0.873
IT systems on my farm make decisions autonomously.	0.454	2.25	1.152
Factor 7: Facilitating conditions (Cronbach’s Alpha: 0.728)			
There is internet access or mobile internet connection on the whole farm.	0.860	3.46	1.461
I meet all the technical requirements to use smart products in a targeted manner (e.g., internet everywhere on the farm).	0.785	3.36	1.281

Kaiser–Meyer–Olkin (KMO) criteria = 0.930; declared total variance = 59.03 percent. All statements were scored with a scale from 1 = “totally disagree” to 5 = “totally agree”. n = 422.

**Table 2:** Results of the cluster analysis.

Factor and Statements	Cluster A (n = 96)	Cluster B (n = 65)	Cluster C (n = 157)	Cluster D (n = 104)
Animal benefits ***	0.27 ^d^ (0.777)	0.57 ^d^ (0.715)	0.32 ^d^ (0.620)	−1.09 ^abc^ (0.988)
Animals can benefit from smart products because deviations from normal behavior are detected more quickly. ***	4.33 ^d^ (0.706)	4.23 ^d^ (0.825)	4.38 ^d^ (0.583)	3.12 ^abc^ (0.988)
Animals can benefit from smart products because diseases are detected more quickly. ***	4.19 ^d^ (0.812)	4.31 ^d^ (0.789)	4.26 ^d^ (0.726)	3.07 ^abc^ (1.026)
I would have a smart product assist me with animal control. ***	4.32 ^d^ (0.900)	4.32 ^d^ (0.812)	4.47 ^d^ (0.656)	2.92 ^abc^ (1.129)
I am pleased when smart products can inform me about the condition of my animals in real time. ***	4.32 ^d^ (0.900)	4.58 ^d^ (0.659)	4.55 ^d^ (0.655)	3.28 ^abc^ (1.194)
Social environment ***	−1.03 ^bcd^ (0.752)	−0.08 ^acd^ (0.959)	0.69 ^abd^ (0.628)	−0.04 ^abc^ (0.824)
I think using smart products makes a good impression in society. ***	2.61 ^bcd^ (0.944)	3.25 ^ac^ (1.041)	3.86 ^abd^ (0.763)	3.12 ^ac^ (0.938)
I think society expects me to use smart products. ***	2.25 ^c^ (0.795)	2.62 ^c^ (1.041)	3.43 ^abd^ (0.969)	2.53 ^c^ (1.042)
My social environment (neighbors, colleagues, friends) approves of smart products on my farm. ***	2.68 ^bc^ (0.989)	3.25 ^ac^ (1.016)	3.89 ^abd^ (0.824)	2.69 ^c^ (1.042)
I think that my colleagues will like it if I use smart products on my farm. ***	2.63 ^bcd^ (0.861)	3.20 ^ac^ (1.003)	3.79 ^abd^ (0.832)	3.18 ^ac^ (1.031)
My family is in favor of smart products on my farm. ***	3.59 ^cd^ (0.924)	3.31 ^c^ (1.089)	4.20 ^abd^ (0.755)	3.09 ^ac^ (0.946)
Effort ***	0.06 ^b^ (0.967)	−0.57 ^acd^ (0.840)	0.02 ^b^ (0.950)	0.28 ^b^ (1.063)
Learning how to use smart products is difficult for me. ***	2.36 ^d^ (1.027)	2.02 ^cd^ (0.992)	2.38 ^d^ (1.034)	2.38 ^abc^ (1.178)
Safe handling of smart products is not easy for me. **	2.77 (1.192)	2.29 ^d^ (1.169)	2.64 (1.160)	2.90 ^b^ (1.153)
I imagine the operation of smart products to be difficult. ***	2.73 ^d^ (0.946)	2.40 ^d^ (0.965)	2.67 ^d^ (0.916)	3.28 ^abc^ (1.038)
Using smart products is a stressor for me. ***	2.39 ^d^ (1.050)	1.97 ^d^ (0.918)	2.24 ^d^ (1.032)	3.10 ^abc^ (1.084)
Trust ***	0.38 ^cd^ (0.922)	0.36 ^cd^ (0.872)	−0.02 ^abd^ (0.857)	−0.55 ^abc^ (1.096)
I am confident that machines work the way they are programmed. ***	3.92 ^d^ (0.816)	3.74 ^d^ (0.853)	3.68 ^d^ (0.734)	3.17 ^abc^ (1.009)
I trust smart products and the decisions they make. ***	3.50 ^d^ (0.834)	3.32 ^d^ (0.831)	3.56 ^d^ (0.737)	2.63 ^abc^ (0.926)
In case of purchasing new smart products, I will get enough information from the manufacturer to use them reasonably. ***	3.69 ^d^ (0.898)	4.00 ^d^ (0.866)	3.66 ^d^ (0.918)	3.08 ^abc^ (1.031)
I think my data are safe when I work with smart products from reputable manufacturers. ***	2.98 ^cd^ (1.187)	3.38 ^d^ (1.071)	3.43 ^ad^ (0.907)	2.48 ^abc^ (0.985)
I would follow a recommendation that a smart product gives me. ***	3.72 ^bd^ (0.706)	3.34 ^ac^ (0.796)	3.66 ^bd^ (0.694)	3.16 ^ac^ (0.777)
Technology readiness ***	0.64 ^bcd^ (0.684)	−1.45 ^acd^ (0.935)	0.30 ^abd^ (0.650)	−0.14 ^abc^ (0.786)
Various seniors are used on my farm. ***	4.84 ^bd^ (0.443)	3.29 ^acd^ (1.343)	4.66 ^bd^ (0.749)	4.11 ^abc^ (0.944)
On my farm, computers are used for certain tasks (e.g., herd management). ***	3.96 ^bd^ (1.123)	2.03 ^acd^ (1.104)	3.91 ^bd^ (1.064)	2.99 ^abc^ (1.195)
I am used to adapting the farm to changing conditions. ***	4.46 ^bd^ (0.739)	3.66 ^ac^ (1.004)	4.46 ^bd^ (0.655)	4.00 ^ac^ (0.655)
IT systems on my farm make decisions autonomously. ***	2.56 ^bd^ (1.113)	1.45 ^ac^ (0.730)	2.74 ^bd^ (1.150)	1.71 ^ac^ (0.889)
Facilitating conditions ***	−0.05 ^c^ (0.994)	−0.47 ^c^ (0.981)	0.44 ^abd^ (0.773)	−0.33 ^c^ (1.062)
There is internet access or mobile internet connection on the whole farm. ***	3.51 ^abd^ (1.384)	2.71 ^ac^ (1.487)	4.15 ^abd^ (1.051)	2.85 ^ac^ (1.563)
I meet all the technical requirements to use smart products in a targeted manner (e.g., internet everywhere on the farm). ***	3.39 ^abd^ (1.243)	2.68 ^ac^ (1.147)	4.01 ^abd^ (1.038)	2.76 ^ac^ (1.235)

Significance level at ** *p* ≤ 0.01; *** *p* ≤ 0.001; letters (a, b, c, d) signify a significant difference to the corresponding cluster (Tamhane post-hoc multiple comparison test at significance level 0.05). Numbers without brackets show mean values, numbers in brackets illustrate the items’ standard derivations. All statements were scored with a scale from 1 = “totally disagree” to 5 = “totally agree”. n = 422.

**Table 3:** Socio-demographic and farm characteristics of the clusters.

	Cluster A (n = 96)	Cluster B (n = 65)	Cluster C (n = 157)	Cluster D (n = 104)
Gender *** (male (female)) ^1^	85 (11) ^b^	33 (32) ^ac^	120 (37) ^b^	81 (23)
Age Ø *** ^1^	44.22 ^b^	35.03 ^acd^	42.96 ^b^	43.71 ^b^
Hectares Ø *** ^1^	474.87^b^	141.88 ^ac^	507.97 ^bd^	228.38 ^c^
Occupation *** (main occupation (secondary occupation)) ^1^	92 (4) ^b^	48 (18) ^acd^	147 (10) ^b^	95 (9) ^b^
Work Experience Ø *** ^2^	25.07 ^b^	15.09 ^acd^	23.06 ^b^	23.92 ^b^
Meat chicken farm (yes (no)) ^1^	7 (89)	3 (62)	10 (147)	7 (97)
Layers (yes (no)) ^1^	8 (88)	10 (55)	20 (137)	18 (86)
Piglet breeding (yes (no)) ^1^	14 (82)	1 (64)	18 (139)	13 (91)
Pig fattening (yes (no)) ^1^	29 (67)	11 (54)	52 (105)	40 (64)
Sows (yes (no)) * ^1^	17 (79) ^b^	1 (64) ^acd^	22 (135) ^b^	18 (86) ^b^
Dairy cattle (yes (no)) * ^1^	48 (48) ^d^	27 (38)	85 (72) ^d^	33 (71) ^ac^
Beef cattle (yes (no)) ^1^	22 (74)	25 (40)	31 (126)	39 (65)
Horses (yes (no)) ** ^1^	10 (86) ^b^	16 (49) ^ac^	12 (145) ^b^	11 (93)
Behavioral intention *** ^2^				
I would use smart products on my farm immediately. *** (µ (σ))	3.58 ^cd^ (0.879)	3.42 ^cd^ (1.044)	3.97 ^abd^ (0.847)	3.01 ^abc^ (1.066)
I plan to use smart products on my farm in the future. *** (µ (σ))	3.90 ^bd^ (0.876)	3.02 ^ac^ (1.082)	4.04 ^bd^ (0.922)	3.06 ^ac^ (1.205)
I think I would interact with smart products during the first few days of use on my farm. *** (µ (σ))	4.25 ^d^ (0.725)	4.51 ^d^ (0.725)	4.42 ^d^ (0.661)	3.88 ^abc^ (1.109)
I would use smart products if there were benefits for animal health, animal welfare, animal behavior, or hygiene conditions in the barn. *** (µ (σ))	4.63 ^d^ (0.567)	4.60 ^d^ (0.725)	4.67 ^d^ (0.571)	3.90 ^abc^ (1.010)
I intend to use smart products on my farm in the near future. *** (µ (σ))	3.81 ^bd^ (0.977)	3.00 ^ac^ (1.237)	4.01 ^bd^ (0.873)	2.84 ^ac^ (1.191)
Use Behavior *** ^1^				
I already use smart products at my farm. *** (yes (no))	79 ^bd^ (17)	25 ^ac^ (40)	123 ^bd^ (34)	54 ^ac^ (50)
I use a smartphone to monitor or control operational units such as machines or livestock facilities, and to fulfill documentation requirements or recruit personnel (operational purposes except phone calls and other communications). *** (µ (σ)) ^3^	3.95 ^ad^ (1.191)	2.58 ^ac^ (1.520)	3.88 ^bd^ (1.205)	2.88 ^ac^ (1.416)
I use small machines or devices in the stables, on machines or in the field that perform and document their tasks without human intervention. *** (µ (σ)) ^3^	2.93 ^bd^ (1.643)	1.85 ^ac^ (1.349)	2.99 ^bd^ (1.540)	1.95 ^ac^ (1.361)

Letters (a, b, c, d) demonstrate a significant difference to the corresponding cluster. Level of significance: * = *p* ≤ 0.05, ** = *p* ≤ 0.01, *** = *p* ≤ 0.001. ^1^ Chi-square test according to Pearson. Pairwise comparison using Bonferroni correction. ^2^ These statements were scored with a scale from 1 = “totally disagree” to 5 = “totally agree”. ^3^ Use behavior was measured by use frequency on a five-point scale from 1 = “never” to 5 = “several times a day.” Tamhane post-hoc multiple comparisons tests at significance level 0.05. n = 422.

## Data Availability

The utilized questionnaire is accessible by contacting the corresponding author.
